# The Association Between Lipoprotein(a) and Atherosclerotic Cardiovascular Disease Severity

**DOI:** 10.31083/RCM46656

**Published:** 2026-04-21

**Authors:** Li Zha, Di Wang, Fangfang Li, Ying Wang, Zhenyu Wang, Kangde Zeng, Shujun Yan, Danyang Liu, Degen Pei, Yue Cao, Yang Yu, Ling Weng, Enze Jin

**Affiliations:** ^1^Department of Cardiovascular Medicine, The Fourth Affiliated Hospital of Harbin Medical University, 150081 Harbin, Heilongjiang, China

**Keywords:** lipoprotein(a), atherosclerotic cardiovascular disease, Gensini score

## Abstract

**Background:**

Lipoprotein(a) (Lp(a)) has emerged as an independent determinant of risk among the multiple factors associated with atherosclerotic cardiovascular disease. This study, which utilized data from the northern Chinese population, aimed to investigate the association between Lp(a) and conventional coronary heart disease risk factors. Furthermore, the Lp(a) level may reflect the severity of vascular stenosis in individuals affected by coronary heart disease, offering valuable insights for future clinical interventions.

**Methods:**

A total of 778 individuals who underwent coronary angiography and were later confirmed to have coronary artery disease from September 2022 to December 2024 participated in this study. Baseline clinical information collected for each participant included sex, age, height, weight, smoking and drinking habits, history of hypertension, diabetes status, lipid parameters, and other pertinent medical characteristics.

**Result:**

The analysis demonstrated that progressive increases in circulating Lp(a) levels were associated with a pronounced escalation in vascular stenosis, a pattern reaching statistical significance (*p* < 0.05). An evaluation of the receiver operating characteristic (ROC) curve indicated that Lp(a) yielded an area under the curve (AUC) value of 0.673 (95% confidence interval (CI): 0.630–0.716; *p* < 0.001) for identifying severe coronary artery stenosis. A significant correlation (r = 0.306; *p* < 0.0001) was revealed in the assessment of the correlation between Lp(a) and the Gensini score. An independent association was observed between Lp(a) levels and the number of diseased coronary arteries (odds ratio (OR) = 1.029, 95% CI: 1.017–1.041; *p* < 0.001). Furthermore, individuals with higher Gensini scores exhibited significantly increased Lp(a) levels. Notably, patients with chronic total occlusion (CTO) lesions or multi-vessel disease also demonstrated markedly higher Lp(a) levels (all *p* values < 0.001).

**Conclusion:**

Elevated Lp(a) concentrations are linked to increased severity of coronary heart disease, as evidenced by higher Gensini scores. Elevated Lp(a) concentrations are also associated with an increased occurrence of multi-vessel coronary artery stenosis or total occlusions.

## 1. Introduction

Owing to its strong genetic determination, lipoprotein(a) exerts an independent influence on the risk of atherosclerotic cardiovascular disease [1]. It is primarily synthesized in the liver and can interact with vascular endothelial cells and cellular receptors, leading to impaired vascular endothelial barrier function. Within the low-density lipoprotein (LDL) variant Lp(a), the core apolipoprotein B100 is covalently connected to apolipoprotein(a) through disulfide linkages, thereby establishing a hydrophilic surface that encapsulates the molecule. The Lp(a) gene, located on the long arm of chromosome 6 at 6q2.6–2.7, encodes apolipoprotein(a) [2]. Although the precise physiological role of lipoprotein(a) remains undefined, the evolutionary resemblance between apolipoprotein(a) and plasminogen implies a potential anti-fibrinolytic function and involvement in prothrombotic pathways. Lp(a) may promote the development of a vulnerable plaque phenotype, thereby accelerating the occurrence of atherosclerotic thrombotic events [3, 4]. Furthermore, it promotes the expression of pro-inflammatory cytokines and induces cell apoptosis [5, 6]. Collectively, these pathways lead to endothelial dysfunction and inflammatory responses, which play a central role in the pathogenesis and advancement of atherosclerosis, a core mechanism underlying cardiovascular injury.

Elevated Lp(a) demonstrated an independent association with a higher likelihood of acute myocardial infarction in a Chinese cohort, even when LDL concentrations remained within the normal physiological range. In South Asian and Latino cohorts, elevated Lp(a) was likewise linked to an increased likelihood of myocardial infarction, independent of classic cardiovascular risk contributors [7, 8]. Genetic profiles from a large population dataset indicated that the Lp(a) locus shows the most pronounced association with coronary artery disease, supported by variation analyses involving more than sixty thousand cases and over one hundred thirty thousand controls [9]. The LipidCardio study, a cross-sectional observational analysis, demonstrated that the group with raised Lp(a) levels had greater SYNTAX-I and Gensini scores [10]. In a different large-scale cross-sectional investigation, Lp(a) demonstrated an independent association with high Gensini scores (≥100), left main coronary artery lesions, and three-vessel coronary disease [11].

Owing to the complex molecular structure of Lp(a), particularly its polymorphic characteristics, the determination of its circulating levels has remained a significant challenge. Persistent variability in reproducibility among analytical approaches has underscored the need for standardized reference materials, a harmonization effort formally advanced by the IFCC to promote greater methodological alignment [12]. Recently, a novel immunoturbidimetric assay utilizing a five-point calibration approach exhibited minimal measurement bias for apo(a) allele size and quantifies Lp(a) in nmol/L [13, 14]. Nevertheless, the clinical application of Lp(a) continues to face challenges, including the lack of universally accepted critical thresholds for diverse global populations [15, 16]. Understanding lipoprotein(a) may provide essential guidance for the proactive management of traditional risk factors. Across six randomized trials comprising 444 participants, consolidated evidence indicated that treatment with mipomersen produced marked reductions in LDL-C, non-HDL-C, apolipoprotein B, and Lp(a) [17]. Network meta-analysis indicated that different types and doses of statins had no significant effect on Lp(a) levels [18]. Similarly, ezetimibe treatment had no significant effect on plasma Lp(a) [19]. In a meta-analysis of 14 randomized controlled trials (RCTs), niacin has been shown to reduce lipoprotein(a) levels [20]. Notably, an additional ~15% reduction in plasma Lp(a) levels was observed when a PCSK9 inhibitor was added to ongoing niacin therapy [21]. This study, utilizing data from the northern Chinese population, aims to investigate the association between Lp(a) and conventional coronary heart disease risk factors. Furthermore, this method explores the relationship between Lp(a) concentrations and the degree of coronary artery stenosis, contributing meaningful information for subsequent clinical applications.

## 2. Methods

### 2.1 Study Population

This study was conducted with support from the Outstanding Young Project at the Fourth Affiliated Hospital of Harbin Medical University. Between September 2022 and December 2024, a total of 778 patients diagnosed with coronary artery disease (CAD), defined by coronary angiography showing ≥50% stenosis, were enrolled. Patients with a prior history of lipid-lowering therapy, including statins or PCSK9 inhibitors, were strictly excluded from participation in this study. Participants were required to meet the following inclusion criteria: (1) Adults aged 18 years or older; (2) Individuals who underwent coronary angiography according to the study protocol. The exclusion criteria comprised: (1) Patients with contraindications for coronary angiography or those unable to participate in vascular function testing; (2) Patients with acute infections, severe arrhythmias, pregnancy or lactation, or significant blood and endocrine system disorders; (3) Patients with incomplete clinical records or who did not undergo coronary angiography. The basic clinical data for each patient included gender, age, height, weight, smoking status, alcohol consumption, history of hypertension, presence of diabetes, lipid profiles, and other relevant medical backgrounds.

The severity of coronary artery stenosis was quantified using the Gensini scoring system, which calculates scores by multiplying specific lesion segments by their corresponding coefficients. The overall total of the values from all segments forms the final score. **Supplementary Table 1** provides detailed scores and associated coefficients for each vessel. CAD was classified as follows (1) Mild CAD ≤25, 25< Moderate CAD ≤50, Severe CAD >50. A 2020 study from J Am Heart Assoc divided Lp(a) into high and medium-low groups based on a threshold of 30 mg/dL [22]. (2) Low-Lp(a) ≤30 mg/dL, High-Lp(a) >30 mg/dL.

### 2.2 Measurement of Lp(a)

The level of Lp(a) was determined by latex-enhanced immunoturbidimetry. The reagent kit was provided by Shanghai Huizhong Biotechnology, China (CZ011) Co., Ltd. The approval number is National Medical Device Import 20152401845. Main components: R1: PBS buffer solution, R2: latex particle suspension; R1: glycine buffer solution, R2: anti-human Lp(a)-IgG sensitized latex; R1: Tris buffer solution, PEG6000, R2: anti-Lp(a) monoclonal antibody sensitized latex; R1: glycine buffer solution, bovine serum albumin, R2: Lp(a) antibody sensitized particles. The within-batch coefficient of variation of the latex-enhanced immunoturbidimetric method is less than 5.5%.

Sample addition: Take a microcentrifuge tube and add each component as shown in the table below:

Additives: Blank tube, Reagent 1 (R1): 240 µL, Distilled water: 5 µL.

Measuring tube, Reagent 1 (R1): 240 µL, Sample: 5 µL.

(1) Incubation: After thorough mixing, incubate at 37 °C in a constant temperature device for 3–5 minutes.

(2) Add Reagent Two: Add 60 µL of Reagent Two (R2) to each tube respectively, and mix well.

(3) Reading: Continue incubating at 37 °C. Read the absorbance A1 30 seconds after adding R2 and then read the absorbance A2 after an exact reaction time of 300 seconds.

(4) Calculation: Calculate the absorbance change value ΔA = A2 – A1. The fully automatic biochemical analyzer (BACKMAN COULTER, Suzhou, China, AU5800) will automatically process the data and calculate the concentration of Lp(a) in the sample based on the calibration curve.

### 2.3 Statistical Analysis

Data analysis was conducted utilizing R software (R Core Team,4.2.0, 2022, Vienna, Austria). Categorical variables within the baseline characteristics of the study participants were displayed as frequencies and proportions. To assess whether data were normally distributed, the Kolmogorov-Smirnov test was applied. The presentation of continuous data was determined by their distribution: normally distributed variables are expressed as mean ± SD, whereas non-normally distributed variables are reported as median and interquartile range. Comparisons across groups were performed using the Kruskal-Wallis non-parametric test. The Spearman rank correlation test was utilized to evaluate the relationship between Lp(a) and other risk factors for coronary heart disease. The diagnostic precision of Lp(a) in identifying coronary artery stenosis was examined through ROC curve analysis, where the area under the curve (AUC) acted as an indicator of its predictive capability.

## 3. Result

### 3.1 The Key Clinical Characteristics of the Study Cohort

A total of 778 patients with coronary atherosclerosis were enrolled in this study. The study cohort was divided into three groups according to the severity of coronary artery stenosis, as determined by quantitative evaluation methods. Table 1 provides an overview of the baseline characteristics for the respective groups. Analysis revealed that patients with higher HDL levels tended to exhibit milder coronary stenosis, with statistical significance (*p* < 0.05). In contrast, elevated levels of low-density lipoprotein cholesterol (LDL) and Lp(a) were strongly correlated with more severe stenosis (*p* < 0.05). Furthermore, patients diagnosed with hypertension, diabetes, or obstructive coronary artery lesions (CTO) exhibited significantly greater vascular narrowing (*p* < 0.05). Notably, individuals with single-vessel disease showed milder stenosis compared to those with multi-vessel disease (*p* < 0.05).

**Table 1.** **Baseline characteristics of patients with coronary artery disease (CAD)**.

	Mild CAD (n = 379)	Moderate CAD (n = 200)	Severe CAD (n = 199)	*p* value
Male, n (%)	226 (59.63%)	130 (65.00%)	127 (63.82%)	0.378
Age, years	64.00 (58.00, 69.00)	64.50 (57.00, 70.00)	65.00 (58.00, 71.00)	0.323
BMI	24.71 ± 3.49	24.85 ± 3.50	25.16 ± 3.35	0.202
Cr, umol/L	69.30 (59.40, 80.60)	71.85 (60.58, 83.98)	70.60 (60.30, 85.60)	0.153
TC, mmol/L	4.72 ± 1.27	4.67 ± 1.16	4.88 ± 1.46	0.495
TG, mmol/L	1.53 (1.14, 2.28)	1.49 (1.09, 2.16)	1.59 (1.12, 2.25)	0.600
HDL, mmol/L	1.04 (0.91, 1.21)	1.00 (0.87, 1.16)	0.98 (0.84, 1.10)	<0.001
LDL, mmol/L	2.61 ± 0.94	2.72 ± 0.95	2.86 ± 1.10	0.044
Lp(a), mg/dL	13.50 (7.50, 24.20)	16.55 (8.40, 33.90)	25.40 (14.20, 46.80)	<0.001
Hypertension, n (%)	211 (55.67%)	123 (61.50%)	132 (66.33%)	0.040
Diabetes, n (%)	62 (16.36%)	54 (27.00%)	60 (30.15%)	<0.001
Smoker, n (%)	148 (39.05%)	81 (40.50%)	75 (37.69%)	0.847
Gensini score	10.00 (5.00, 18.00)	38.00 (32.00, 44.00)	65.00 (56.00, 80.00)	<0.001
CTO, n (%)	0 (0%)	31 (15.50%)	99 (49.75%)	<0.001
Single-vessel disease, n (%)	194 (51.19%)	27 (13.50%)	13 (6.53%)	<0.001

Data are presented as IQR, mean ± SD, or n (%). Abbreviations: CAD, coronary artery disease; Cr, Creatinine; TC, Total cholesterol; TG, triglycerides; HDL, high-density lipoprotein cholesterol; LDL, low-density lipoprotein cholesterol; Lp(a), lipoprotein(a); CTO, obstructive coronary artery lesions.

### 3.2 Investigation of the Statistical Relationship Between Lp(a) Levels and the Severity of Coronary Artery Stenosis

**Fig. 1.**
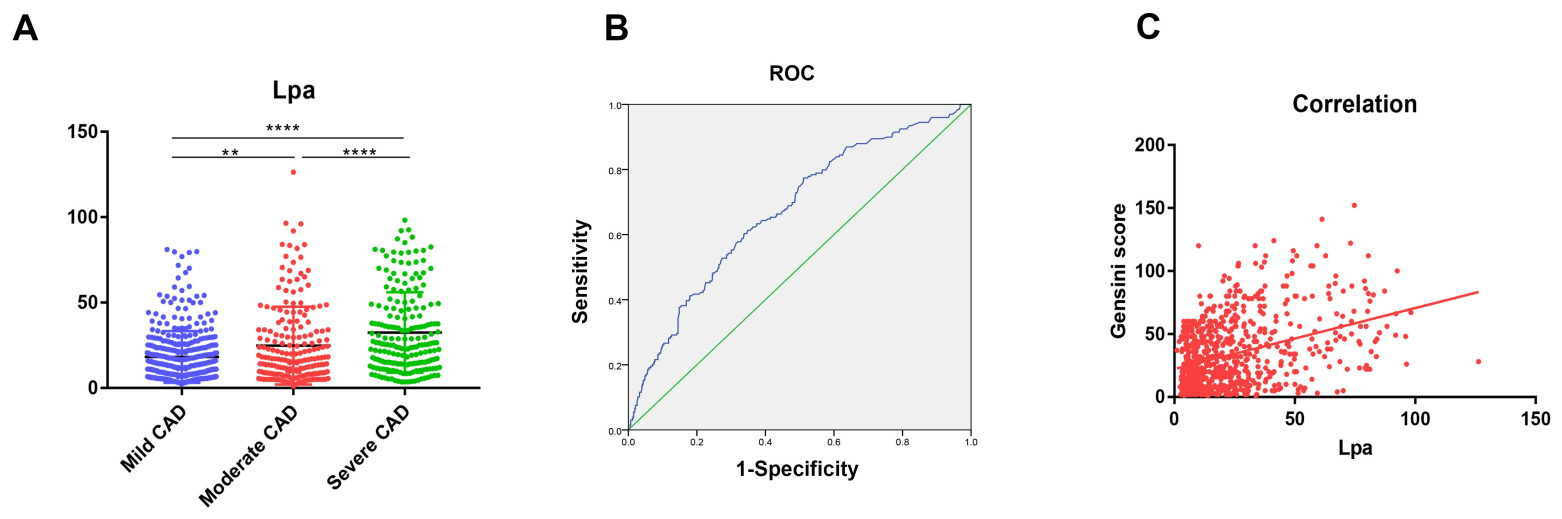
**The levels of Lp(a) and correlations in patients with different degrees of coronary artery stenosis**. (A) Mild, Moderate and Severe group was shown in Dot plot. ** *p* < 0.01, **** *p* < 0.0001. (B) ROC curve analysis. (C) The correlation between Lp(a) and Gensini scores was assessed using Spearman’s method. Receiver Operating Characteristic curve (ROC).

The Lp(a) levels of patients were evaluated across groups categorized by varying degrees of coronary artery stenosis, followed by inter-group comparisons. Statistically significant differences were identified between the mild and moderate stenosis groups, the moderate and severe stenosis groups, as well as the mild and severe stenosis groups (*p* < 0.05) (Fig. 1A). Using ROC curve analysis, the AUC for Lp(a) in patients with severe coronary artery stenosis was determined to be 0.673 (95% confidence interval (CI): 0.630–0.716, *p* < 0.001) (Fig. 1B). To examine the association between Lp(a) and Gensini score, a Spearman correlation analysis was conducted, revealing a significant correlation (r = 0.306, *p* < 0.0001) (Fig. 1C). As presented in Table 2, an analysis was conducted to evaluate the correlation between Lp(a) and risk factors associated with coronary heart disease. The results demonstrated that Lp(a) levels were significantly influenced by age, total cholesterol, and LDL-C (*p* < 0.05).

**Table 2.** **Correlation between the Lp(a) and the CAD risk factors**.

	Correlation R	*p* value
Age, years	0.097	0.007
TC, mmol/L	0.137	<0.001
TG, mmol/L	–0.054	0.133
HDL, mmol/L	–0.012	0.743
LDL, mmol/L	0.203	<0.001
Gender	–0.009	0.796
Hypertension	0.002	0.964
Diabetes	0.002	0.945
Smoker	–0.017	0.645

Abbreviations: TC, Total cholesterol; TG, triglycerides; HDL, high-density lipoprotein cholesterol; LDL, low-density lipoprotein cholesterol.

### 3.3 The Prognostic Importance of Lpa in Assessing the Severity of Coronary Artery Disease

This study applied multivariate logistic regression analysis to examine the relationship between Lp(a) and the severity of coronary artery stenosis in patients with coronary heart disease (Table 3). Analyses indicated that elevated Lp(a) levels were significantly linked to severe coronary artery stenosis, with an odds ratio of 1.036 (95% confidence interval: 1.026–1.046, *p* < 0.001). After adjusting for potential confounding factors, including hypertension, diabetes, LDL, HDL, CTO lesions, and single-vessel disease, Lp(a) significantly increased the likelihood of lesioned vessels (odds ratio [OR] 1.029, 95% confidence interval [CI]: 1.017–1.041, *p* < 0.001).

**Table 3.** **Association between Lp(a) and CAD Severity**.

	Model A		Model B		Model C	
	OR (95% CI)	*p* value	OR (95% CI)	*p* value	OR (95% CI)	*p* value
Mild CAD	1		1		1	
Moderate CAD	1.021 (1.011, 1.031)	<0.001	1.022 (1.012, 1.031)	<0.001	1.015 (1.005, 1.026)	0.005
Severe CAD	1.036 (1.026, 1.046)	<0.001	1.037 (1.027, 1.046)	<0.001	1.029 (1.017, 1.041)	<0.001

Model A: unadjusted; Model B: controlled for hypertension and diabetes; Model C: controlled for diabetes, hypertension, HDL, LDL, single-vessel disease, and CTO. Abbreviations: CI, confidence interval; OR, odds ratio; TG, triglycerides; HDL, high-density lipoprotein cholesterol; LDL, low-density lipoprotein cholesterol; CTO, chronic total occlusion.

### 3.4 Correlation Between Lp(a) Concentrations and the Severity of Coronary Stenosis Resulting From Arterial Lesions

As detailed in Table 4, patient grouping was performed according to Lp(a) levels, in accordance with the methodology described earlier. The baseline characteristics of these groups are presented in Table 4. In individuals with higher Lp(a) levels, elevations in total cholesterol and low-density lipoprotein cholesterol were concurrently observed. Furthermore, individuals with higher Gensini scores exhibited significantly increased Lp(a) levels. Notably, patients with chronic total occlusion (CTO) lesions or multi-vessel disease also demonstrated markedly higher Lp(a) levels (all *p* values < 0.001).

**Table 4.** **Characteristics of CAD patients**.

	Low-Lp(a) (n = 579)	High-Lp(a) (n = 199)	*p* value
Male, n (%)	357, 61.66%	126, 63.32%	0.678
Age, years	64.00 (57.00, 70.00)	64.00 (57.00, 70.00)	0.603
BMI	25.00 ± 3.47	24.46 ± 3.39	0.065
Cr, umol/L	70.10 (60.10, 80.90)	71.20 (60.00, 90.30)	0.098
TC, mmol/L	4.64 ± 1.23	5.07 ± 1.43	<0.001
TG, mmol/L	1.54 (1.12, 2.27)	1.42 (1.12, 2.16)	0.266
HDL, mmol/L	1.01 (0.88, 1.17)	1.02 (0.88, 1.17)	0.858
LDL, mmol/L	2.59 ± 0.92	3.03 ± 1.10	<0.001
Lpa, mg/dL	11.90 (7.10, 19.40)	47.90 (36.90, 66.80)	<0.001
Hypertension, n (%)	344, 59.41%	122, 61.31%	0.638
Diabetes, n (%)	130, 22.45%	46, 23.12%	0.847
Smoker, n (%)	222, 38.34%	82, 41.21%	0.475
Gensini score	22.00 (8.00, 45.00)	44.00 (22.00, 74.00)	<0.001
CTO, n (%)	79, 13.64%	51, 25.63%	<0.001
Single-vessel disease, n (%)	198, 34.20%	36, 18.09%	<0.001

Data are presented as IQR, mean ± SD, or n (%). Abbreviations: Cr, Creatinine; TC, Total cholesterol; TG, triglycerides; HDL, high-density lipoprotein cholesterol; LDL, low-density lipoprotein cholesterol; Lp(a), lipoprotein(a); CTO, obstructive coronary artery lesions.

## 4. Discussion

It was observed that Lp(a) levels varied markedly with the complexity of coronary artery lesions, as evaluated by the Gensini score, in patients with coronary atherosclerotic heart disease. Specifically, individuals with a Gensini score greater than 50 show markedly elevated Lp(a) concentrations. Additionally, when Lp(a) exceeds 30 mmol/L, the severity and progression of coronary artery disease were notably exacerbated. The current literature provides a comprehensive account of Lp(a)’s role in atherosclerosis, yet global standardization of its measurement methods remains inadequate, limiting the precise identification of high-risk individuals. Furthermore, existing evidence supporting the clinical benefits of reducing Lp(a) levels is limited. It is expected that emerging therapeutic approaches in the coming years will provide innovative solutions for managing this condition [23].

A 2022 Consensus Statement by the European Atherosclerosis Society critically reviewed contemporary evidence on the involvement of Lp(a) in atherosclerotic cardiovascular disease. The 2022 Consensus Statement delivered current recommendations for detecting and managing elevated Lp(a) and evaluated the possibility of integrating it into comprehensive cardiovascular risk assessments. Evidence further establishes Lp(a) as a pathogenic factor contributing to adverse cardiovascular outcomes. Ongoing and future clinical trials specifically targeting Lp(a) reduction are deemed critical for establishing its clinical utility and therapeutic value in the prevention and management of cardiovascular disease [24]. Ethnic background may impact Lp(a) concentrations. Analysis of 460,506 middle-aged individuals from the UK Biobank cohort revealed a significant positive relationship between Lp(a) concentrations and the incidence of atherosclerotic cardiovascular disease, with the study population exhibiting a median Lp(a) level of 19.6 nmol/L [25]. Additionally, the 2017 European prospective cohort study assessed Lp(a) concentrations in over 52,000 participants, with a median value of approximately 21 nmol/L [26]. This pattern is clearly reflected in the UK Biobank dataset, which demonstrates a progressively increasing median concentration of Lp(a) among distinct ethnic populations, following the order of Chinese, White, South Asian, and Black individuals, with respective measured levels of 16, 19, 31, and 75 nmol/L [27].

In this research, patients were classified into three groups according to the severity of their coronary heart disease. The median lipoprotein(a) levels in those with mild, moderate, and severe disease were recorded at 13.5, 16.55, and 25.4 mg/dL (equivalent to roughly 34.8, 42.8 and 65.6 nmol/L), respectively. Overall, the median lipoprotein(a) concentration across all study participants was 16.9 mg/dL, or approximately 43.6 nmol/L. The data indicate that participants in our cohort exhibited relatively elevated Lp(a) levels compared to previous reports. One possible explanation for this discrepancy could be the ethnic homogeneity of our study population, as all participants were of Han Chinese descent. Research has demonstrated that certain East Asian populations commonly exhibit an increased number of Kringle IV type 2 repeat copies within the Lp(a) gene, which has been linked to decreased Lp(a) production [28]. Moreover, scientific evidence indicates that various factors—including single nucleotide polymorphisms, epigenetic modifications affecting promoter activity, and other mechanisms—may also contribute to the regulation of Lp(a) levels [29]. While genetic factors chiefly determine Lp(a) levels, other modulatory influences may also contribute, environmental factors may exert a modest modulatory effect in the context of underlying genetic backgrounds. For instance, consumption of diets high in saturated and trans fatty acids has been associated with slight increases in Lp(a) levels. Among individuals diagnosed with hyperlipoproteinemia, the use of L-carnitine, coenzyme Q10, and Xuezhikang corresponded with notable decreases in plasma Lp(a) concentrations [30]. Various natural agents, such as pectin, Ginkgo biloba extract, flaxseed, red wine, resveratrol, and curcumin derivatives, have been observed to exert a limited yet measurable effect in lowering elevated Lp(a) concentrations [31]. Certain chronic medical conditions, such as renal insufficiency, may also induce minor alterations in Lp(a) concentrations [32, 33]. Furthermore, multiple studies have reported that both endogenous elevations and exogenous administration of human growth hormone can lead to increased circulating Lp(a) levels [34, 35]. Our study has several limitations. Comprehensive dietary histories were not obtained from participants, and the use of oral hormonal therapies was not included in the analysis, both of which may influence Lp(a) measurements and potentially contribute to discrepancies when compared with previous investigations. To enhance the generalizability of future findings, it is recommended to conduct multi-center, multi-platform studies incorporating diverse ethnic populations, thereby improving sample heterogeneity and substantially increasing the total sample size.

The pivotal role of Lp(a) in cardiovascular disease development has led to heightened research interest. Elevated concentrations of lipoprotein(a) are closely linked to a heightened risk of aortic valve calcification. Evidence suggests that overexpression of the Lp(a) gene may promote this pathological process, potentially through mechanisms such as disruption of the transforming growth factor-β signaling pathway [36]. The pro-inflammatory and atherogenic activities of Lp(a) appear to be partly driven by the oxidized phospholipids associated with the particle [24]. According to a Mendelian randomization study, the association of Lp(a) with coronary heart disease events is several-fold stronger than that of low-density lipoprotein cholesterol [37]. Another study has shown that Lp(a) exerts a more significant influence on future cardiovascular risk when present at concentrations exceeding the 90th percentile [38]. Evidence suggests that individuals with increased Lp(a) concentrations (e.g., ≥150 nmol/L) demonstrate a higher incidence of coronary artery disease and a greater likelihood of presenting with diffuse vascular stenosis [10]. In this cohort, Lp(a) concentrations were categorized into low and high groups. The high-Lp(a) group demonstrated a lower incidence of single-vessel disease and a higher prevalence of coronary occlusive lesions. Furthermore, higher Lp(a) molar levels demonstrated a significant relationship with both the occurrence and the clinical severity of coronary artery disease. Disease severity was operationally defined as the presence of multi-vessel disease and higher Gensini scores, which reflect the overall burden of coronary stenosis [39]. In our investigation, the Gensini score was significantly higher among individuals with elevated Lp(a) levels, with a median value of 44. A stepwise rise in Lp(a) concentrations was observed corresponding to escalating coronary artery disease severity, with the trend most pronounced across mild, moderate, and severe classifications based on Gensini scoring. These findings are consistent with those of prior investigations. Notably, a cohort study conducted in China, which included 6714 consecutive patients undergoing percutaneous coronary intervention, revealed that Lp(a) concentrations were strongly correlated with the severity of coronary artery disease and could independently predict elevated SYNTAX scores [40]. In a single-center prospective study of 774 patients with acute coronary syndrome, the median baseline Lp(a) level was 21.85 mg/dL, showing a positive correlation with both the Gensini and SYNTAX scores [41]. In our study, only the correlation between Lp(a) and Gensini score was compared, which was positively correlated with an r value of 0.306. Our research partially shares the same trend as this study, but the correlation is slightly stronger. However, our AUC area is 0.67, which is lower than that of this study. This indicates that although Lp(a) in our model is a better predictor than random guessing, its performance has not yet reached a good level and is of moderate to lower discriminatory ability, with a certain degree of resolution. Analysis of our cohort indicated that Lp(a) functioned as a minor risk factor for severe coronary stenosis, with an odds ratio of 1.029; each 1 mg/dL elevation in Lp(a) was associated with a roughly 2.9% higher risk, suggesting a limited effect magnitude. The risk magnitude found in this study is lower than that reported in several large meta-analyses and cohort studies. Discrepancies observed within the study cohort could be attributed to methodological differences in Lp(a) assessment, the extent of adjustment for confounding factors, and the inherently genetic nature of Lp(a) combined with substantial variability among individuals. When it is treated as a continuous variable, the distribution range may be relatively narrow, thereby limiting the ability to detect a stronger association. Lp(a) may be incorporated into a larger risk prediction model and used in combination with other indicators, potentially contributing some predictive value to the overall model.

## 5. Limitations

In our present study, only the Gensini score was employed as the primary evaluation metric, while the SYNTAX score was not included. This limitation will be addressed in future research. Several additional limitations should also be acknowledged. Lp(a) levels were assessed at a single time point, and potential fluctuations over time were not considered, which may have influenced the study outcomes. Moreover, although adjustments were made for recognized confounding factors, the possibility of residual confounding cannot be excluded and may affect the reliability of the observed associations. Some important confounding factors such as estimated glomerular filtration rate, high-sensitivity C-reactive protein, and the use of antihypertensive drugs were not included in the multivariate model, which may reduce the specificity and sensitivity of the model’s diagnosis. In our study, each candidate underwent only a single Lp(a) test, which could be affected by the intra-batch and inter-batch coefficient of variation of the reagent kit, the time difference between sample collection and testing, and the storage time at 4 °C, potentially leading to errors in Lp(a) test data. This is a single-center, cross-sectional study. In the future, multi-center and multi-platform candidates should be included to expand the range of participants and increase the generalizability of the predictive value. Future studies will conduct follow-up of patients, and major cardiovascular events will be further included in the follow-up outcomes to enhance the clinical predictive results of the article.

## 6. Conclusion

Elevated concentrations of Lp(a) correspond to greater coronary heart disease severity, as indicated by higher Gensini scores. Individuals exhibiting elevated Lp(a) levels have a greater incidence of multi-vessel coronary stenosis or total occlusions, supporting its importance as an independent contributor to coronary heart disease. Consequently, timely and aggressive management may be crucial in mitigating the risk of cardiovascular complications in this patient group.

## Data Availability

Data and Materials can obtain on website: https://www.scidb.cn/en/s/bARni2.

## Abbreviations

Lp(a), lipoprotein (a); LDL, Low-density lipoprotein; HDL, High-density lipoprotein; CTO, chronic total occlusion; ASCVD, atherosclerotic cardiovascular disease; CAD, Coronary artery disease; Cr, Creatinine; TC, Total cholesterol; TG, triglycerides.

## Supplementary Material

Supplementary material associated with this article can be found, in the online version, at https://doi.org/10.31083/RCM46656.
