# Supplementary material for: The Association Between Lipoprotein(a) and Atherosclerotic Cardiovascular Disease Severity
Source: Rev Cardiovasc Med. 2026 Apr 21;27(4):46656. doi: 10.31083/RCM46656 (PMC13155971; doi:10.31083/RCM46656)
Supplement: Supplementary file 1 [file 2153-8174-27-4-46656-s1.zip › Supplementary Material.docx]

Supplementary Table 1. Gensini score

| Stenosis | score | Lesion | score |
| --- | --- | --- | --- |
| 1%-25% | 1 | LM | 5 |
| 26%-50% | 26 | Proximal LAD or LCX | 2.5 |
| 51%-75% | 4 | Middle LAD | 1.5 |
| 76%-90% | 8 | Distal LAD | 1.0 |
| 91%-99% | 16 | Middle or distal LCX | 1.0 |
| 100% | 32 | RCA | 1.0 |
|  |  | Small branch | 0.5 |

LM, left main; LAD, left interior descend; LCX, left circumflex; RCA, right coronary artery; The Gensini score for each affected segment is equal to the stenosis score multiplied by the lesion factor.
